# The integrin receptor beta_7_ subunit mediates airway remodeling and hyperresponsiveness in allergen exposed mice

**DOI:** 10.1186/s12931-024-02899-8

**Published:** 2024-07-12

**Authors:** Miri Assayag, Tahrir Obedeyah, Avraham Abutbul, Neville Berkman

**Affiliations:** Department of Pulmonary Medicine, Hadassah Medical Center and Faculty of Medicine, Hebrew University of Jerusalem, Ein Kerem, Jerusalem, Israel

**Keywords:** Airway-hyperresponsiveness, α4β7 integrin, Asthma, Fibroblast, Remodeling

## Abstract

**Background:**

Fibroblast differentiation to a myofibroblast phenotype is a feature of airway remodeling in asthma. Lung fibroblasts express the integrin receptor α_4_β_7_ and fibronectin induces myofibroblast differentiation via this receptor.

**Objectives:**

To investigate the role of the β7 integrin receptor subunit and α_4_β_7_ integrin complex in airway remodeling and airway hyperresponsiveness (AHR) in a murine model of chronic allergen exposure.

**Methods:**

C57BL/6 wild type (WT) and β7 integrin null mice (β_7_ -/-) were sensitized (days 1,10) and challenged with ovalbumin (OVA) three times a week for one or 4 weeks. Similar experiments were performed with WT mice in the presence or absence of α_4_β_7_ blocking antibodies. Bronchoalveolar (BAL) cell counts, AHR, histological evaluation, soluble collagen content, Transforming growth factor-β (TGFβ) and Interleukin-13 (IL13) were measured. Phenotype of fibroblasts cultured from WT and β_7_ -/- saline (SAL) and OVA treated mice was evaluated.

**Results:**

Eosinophil numbers were similar in WT vs β7-/- mice. Prolonged OVA exposure in β7-/- mice was associated with reduced AHR, lung collagen content, peribronchial smooth muscle, lung tissue TGFβ and IL13 expression as compared to WT. Similar findings were observed in WT mice treated with α_4_β_7_ blocking antibodies. Fibroblast migration was enhanced in response to OVA in WT but not β7 -/- fibroblasts. α-SMA and fibronectin expression were reduced in β7-/- fibroblasts relative to WT.

**Conclusions:**

The β7 integrin subunit and the α4β7 integrin complex modulate AHR and airway remodeling in a murine model of allergen exposure. This effect is, at least in part, explained by inhibition of fibroblast activation and is independent of eosinophilic inflammation.

**Supplementary Information:**

The online version contains supplementary material available at 10.1186/s12931-024-02899-8.

## Introduction

Airway remodeling (AWR) is defined as changes in size, mass, or number of tissue structural components that occurs in the airways in response to injury and/or chronic inflammation [1] and may be considered physiological or pathological [2, 3]. Remodeling in asthma is considered to be an important contributing factor in the pathophysiology of airway hyperresponsiveness and may account, at least in part, for refractoriness to anti-inflammatory therapy in patients with more severe disease [4–6]. Although AWR includes epithelial injury, basement membrane thickening, mucus gland hypertrophy, and neovascularity, the predominant elements are an increase in airway smooth muscle (ASM) mass and increased extra-cellular matrix (ECM) deposition [1, 2, 7, 8]. AHR has been linked to subepithelial fibrosis, increased number of myofibroblasts and smooth muscle area [9, 10]. We have previously demonstrated in a murine model of chronic allergen-induced airway remodeling that attenuating airway fibrosis, independent of changes in airway inflammation, protects against the development of AHR [11].

Airway fibroblasts synthesize and secrete extra-cellular matrix (ECM) components, such as collagens, glycoproteins and proteoglycans, and ECM-degrading proteases [12]. Fibroblasts demonstrate phenotypic heterogeneity ranging from the non-contractile, quiescent, non-secretory fibroblast to the contractile, proliferative and highly secretory "myofibroblast" [12, 13]. Myofibroblasts are present in abundance in tissues undergoing repair [13] and are increased in airway remodeling in asthma [2, 7].

Integrins are a family of heterodimeric transmembrane cell receptors that bind ECM components and mediate the interaction between cells and their extracellular environment [14–16] including cell migration, wound healing, cell differentiation, adhesion and apoptosis. There are at least 24 heterodimers formed from eight different β subunits and eighteen α subunits. Multiple integrins bind to ligands containing the “RGD” tripeptide (arginine-glycine-aspartate). The α4 subunit is found in association with β1 and β7 and binds to an “LDV” (leucine-aspartate-valine) binding site. α4 null mice die prior to birth with evidence of impaired cardiac development [17]. Integrin β7 associates with the integrin α4 or αE subunits [18]. The α_4_β_7_ integrin receptor complex has previously been reported in human and murine eosinophils, basophils, macrophages, mast cells, NK cells, CD4 ( +) T, B lymphocytes and endothelial cells [19] and its ligands include mucosal addressin cell adhesion molecule-1 (MAdCAM-1) and vascular cell adhesion molecule-1 (VCAM-1) and fibronectin [20]. This integrin plays a role in cell adhesion, rolling, differentiation and survival of eosinophils and lymphocytes [21]. β7 is important for the normal development of Peyer's patches in the intestinal mucosa and β7-deficient mice have impaired recruitment of lymphocytes, mast cell progenitors and eosinophils to the gastrointestinal tract [22]. Several integrins are known to be present on fibroblasts, notably the α_5_β_1_ integrin which binds to the RGD motif of fibronectin and other ECM proteins and stimulates cell adhesion, migration and proliferation [23]. α_4_β_1_ and α_9_β_1_ are also expressed on fibroblasts [24]. We have previously shown that α4β7 is expressed on murine lung fibroblasts and identified it as a key participant in Extra-domain A containing-Fibronectin (EDA-FN) mediated fibroblast differentiation [25]. Expression of EDA-containing fibronectin is enhanced in a murine model of chronic allergen induced airway remodeling and EDA-/- mice have attenuated airway fibrosis following allergen challenge and are protected from developing airway hyperresponsiveness [11].

In the present study, we use both integrin β7 -/- mice and administration of blocking α4β7 integrin antibody *in-vivo*, to demonstrate that this receptor contributes to airway remodeling in a murine model of allergen-induced "asthma". We also demonstrate that activation and differentiation of murine lung fibroblasts from β7 -/- mice are impaired.

## Methods

### Animals

C57BL/6 mice (male and female) were purchased from Harlan Laboratories Ltd (Jerusalem, Israel). β_7_ deficient mice (C57BL/6-Itgb7/J) were purchased from Jackson Laboratories (Bar Harbor, Maine, USA). Absence of β_7_ gene expression was confirmed by PCR from DNA obtained from β_7_ deficient mice (Supplementary Fig. 1). All animals were housed under specific pathogen-free (SPF) conditions, 4–5 mice/ cage (lined with wood shavings), with unlimited access to sterilized chow and water with 12-h light and dark cycle. Experiments are designed using 8–10 animals/group to allow statistical comparisons. Animals were examined three times weekly and weighed twice weekly during the treatment phase of the protocol. Experimental protocol prespecified that mice that lost more than 20% of initial weight or that showed signs of distress or illness would be removed and sacrificed, however none of the mice required early removal. The Hebrew University-Hadassah Medical School Ethics Committee approved all experimental animal protocols (approval no:MD-12–13,218-3, MD-13–13,627-4) and is accredited by the US National Institute of Health (F16-00010 (A5011-01). Protocols were in accordance with ARRIVE guidelines for performance of animal experiments.

### Allergen challenge protocol

**Fig. 1:**
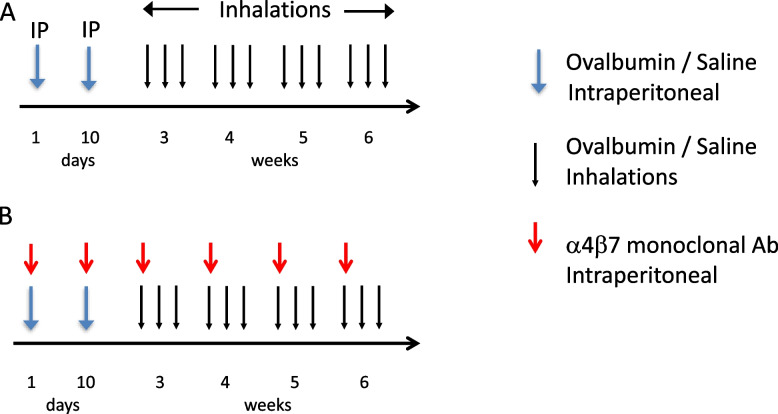
Schematic representation of allergen challenge models. Ten-eleven week old C57BL/6 mice were sensitized with intraperitoneal (IP) ovalbumin (OVA) (10 μg OVA/1 mg Al (OH)_3_ in 0.5 ml 0.9% saline or with saline on days one and 10. Mice were then challenged with inhaled saline or OVA (2% weight/volume diluted in 0.9% saline: 4 ml/inhalation) three times a week for one (“acute”) to 4 weeks (“chronic”), starting on day 15. (A) Allergen challenge using β 7-/- mice or wild-type controls; (B) Mice treated with saline or α_4_β_7_ blocking antibody (DAKT32), 100ug in 100µl / dose administered intraperitoneally weekly for 6 weeks

Figure 1 , Animals were randomized into different treatment groups using excel RAND function**.** Ten-eleven week old mice were sensitized with intraperitoneal OVA (10 μg OVA/1 mg Al (OH)_3_ in 0.5 ml 0.9% saline (Sigma, St. Louis, MO), or with saline on days one and 10. Mice were then challenged with inhaled saline or OVA (2% weight/volume diluted in 0.9% saline: 4 ml/inhalation) three times a week for one to 4 weeks, starting on day 15. For challenge, mice were transferred into a perspex 30 × 40 x 40 cm box, and exposed to saline or OVA administered by means of a micro-mist nebulizer (Hudson RCI, Temecula, CA) with a flow rate of 7L/min for 20 min. For studies using α_4_β_7_ blocking antibody (DAKT32, Biolegend, CA), 100ug in 100µl PBS / dose was administered intraperitoneally weekly for 6 weeks.

### Airway hyperresponsiveness (AHR)

Twenty-four hours after the final allergen challenge, mice were anesthetized with intraperitoneal ketamine/xylasine 20 mg/kg, a metal cannula was inserted in the trachea and mice were attached to a Flexivent ventilator system (Scireq, Montreal). Lung resistance were measured in response to increasing doses of methacholine up to 64 mg/mL [11]. Mice were then sacrificed and broncho-alveolar lavage (BAL) fluid obtained for differential cell counts and enzyme-linked immunosorbent assay (ELISA). Total and differential cell counts from BAL were performed using cytospin and Hematocolor staining. Lung function measurements were performed in a blinded fashion (without knowing what treatment the animal had received).

### Lung pathology

The right lung was cryopreserved for RNA analysis, collagen content determination and tissue ELISA and the left lung for histological staining (hemotoxylin-eosin [H&E], Masson Trichrome or periodic Acid-Shiff (PAS) or immunostaining (immunohistochemistry (IHC) or immunofluorescence (IF). Quantification was performed using Image pro-Plus computer software and according to published recommendations [26].

### Fibroblast isolation and culture

Lungs from OVA- and saline-treated animals were removed, minced and incubated (37 °C, 5% CO_2_) for 45 min in phosphate buffered saline (PBS) containing 1 mg/ml collagenase (Sigma-Aldrich, St Louis, MO). After passage through a dissociation sieve (Sigma), cells were cultivated in “fibroblast medium” consisting of RPMI 1640 (Sigma) with 10% foetal bovine serum (FBS) (Beit Haemek), gentamycin sulfate (Gibco, Grand Island, NY), 2-mercapthoethanol (Sigma), non-essential amino acids (Beit Ha-emek), glutamine (Gibco), antibiotics (Gibco) and indomethacin. Cells were incubated at 37ºC in 5% CO_2_. Cells in passage 2–4 were used for experiments.

### Immunofluorescence

Twenty-five thousand passage 2 lung fibroblasts were cultured on round cover slips placed in 24-well plates until reaching sub-confluence, were fixed (paraformaldehyde), permeabilized (Triton), blocked (BSA solution) and incubated with monoclonal antibodies for alpha-smooth muscle actin (α-SMA) (Sigma) and fibronectin (Abcam) in the presence or absence of murine transforming growth factor beta (TGF-β) 10 ng/ml (R&D systems, Minneapolis, MN). Binding was detected using fluorescein isothiocyanate (FITC) and cyanine-5 (Cy5) -conjugated secondary antibodies (Jackson ImmunoResearch Laboratories, Inc, West Grove, PA).

### Determination of lung collagen content

Soluble lung collagen content was determined using the Sircol Collagen Assay kit (Biocolor Ltd., Belfast).

#### ELISA

Cytokine expression in BAL fluid and lung tissue for TGFβ and IL-13 were performed using Duoset ELISA kits (R&D systems, Minneapolis, MN, or Peprotech, USA).

### Wound scratch assay for evaluating cell migration

Lung fibroblasts were grown to confluency in 12-well plates. A cell-free wound area was then created by scratching the cells with a pipette tip. The time of the scratching wound was designated as time 0. Cells were then allowed to migrate into the cell-free wound for 16 h. Results were expressed as percentage of the recovered wound area.

### Goblet cell number

The number of periodic acid-Schiff (PAS)-positive and PAS-negative bronchial epithelial cells was determined in individual airways. 5 cross-sectional airways were examined for each mouse. A semi-quantitative score was given per bronchus, on a scale of 0–5 (0: no PAS positive cells, 1: < 10%, 2: 10–25%, 3: 25–50%, 4: 50–75%, 5: > 75%). Results were expressed as the average score per mouse.

### Quantification of peribronchial smooth muscle area

The area of peribronchial α-SMA immunostaining was outlined and quantified using an Axiolab light microscope (Zeiss, Oberkochen, Germany), a Coolpix 990 camera (Nikon, Tokyo, Japan), and analized with Image ProPlus (Media Cybernetics, Inc., Silver Spring, MD). Results are expressed as the area of α-SMA stained per μm of basement membrane of bronchi. Five bronchi per mouse were analyzed.

### Statistical analysis

The mean and standard error of mean (SEM) are given for each group. Measurements and calculations were performed with blinding to treatment for measurements and to groups for calculations. Statistical analysis was performed using GraphPad Prism software. Unpaired Student *t* test or two-way Analysis of Variance (ANOVA) followed by Bonferroni posttest were used for sample size of *n* = 8–10. For sample size *n* = 4, group comparisons were performed by the non-parametric Kruskal–Wallis test and two-group comparisons were performed using the Mann–Whitney test. *P* < 0.05 was considered statistically significant. No data points were excluded from analyses.

## Results

### Integrin β7-/- mice

#### Airway inflammation

**Fig. 2:**
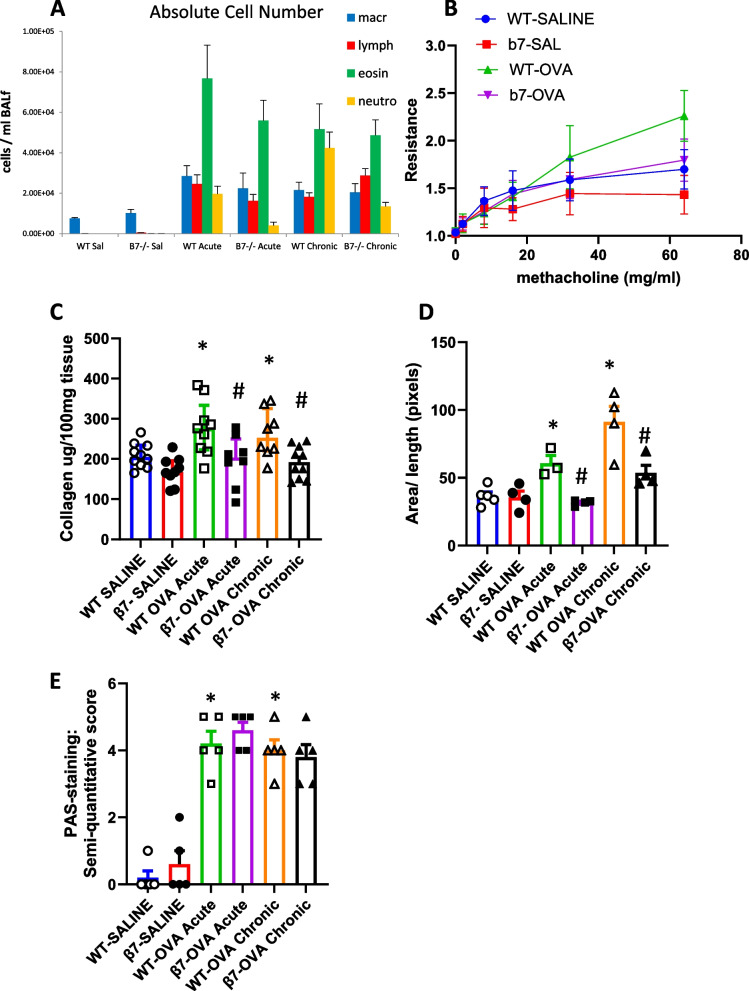
Bronchoalveloar cell counts, airway hyperresponsiveness and airway remodeling in β 7-/- and WT mice. **A** Bronchoalveolar fluid cell counts (per ml) from C57BL/6 wild type (WT) and β 7-/- mice treated with saline or OVA for one ("acute") or four weeks ("chronic"). **B** Airway hyperresponsiveness (resistance—R) in response to increasing doses of inhaled methacholine (MCH) in wild type and β 7-/- mice treated with saline or OVA for four weeks. Resistance at the maximal MCH dose (64 mg/ml). *p* = 0.03 for OVA vs SAL in WT mice, *p* = 0.048 for WT-OVA vs β7 -/- OVA, Area under the curve: *p* = 0.08 WT-OVA vs β7 -/- OVA. N = 8–10 mice per group. **C** Soluble collagen content (ug/100 mg tissue) measured by Sircol in the lungs of acute and chronic OVA treated WT and B7-/- mice. **D** Peribronchial smooth muscle area expressed as pixel number (area/length) using image J Pro Plus software in Masson Trichrome stained lung sections. n = 8–10 mice per group for collagen and 4–5 per group for smooth muscle. **E** Semi-quantitative score for epithelial goblet cells in PAS-stained sections n = 5 mice per group *p < 0.05 vs WT-SAL and vs β7 -/-SAL, #*p* < 0.05 vs WT-OVA acute and vs WT-OVA Chronic

C57BL/6 wild type and β7-/- mice were exposed to SAL or OVA by inhalation for one ("acute") or four ("chronic") weeks. BAL was performed 24 h after the last inhalation. There was a marked increase in total BAL cell counts in response to OVA exposure after one ("acute") and four ("chronic") weeks. OVA was associated with significant increase in number of eosinophils, lymphocytes and neutrophils in both WT and β7-/- mice. Eosinophil numbers were not significantly different in WT vs β7-/- mice (WT-SAL 0 cells/ml, β7-/- 6.3 ± 6; 1w: WT-OVA 76738 ± 16,490, β7-/- OVA 55957 ± 10,022, 4w: WT-OVA 51649 ± 12,495, β7-/- OVA: 48,556 ± 7733, p > 0.05) (Fig. 2A).

### Airway hyperresponsiveness (AHR)

Total lung resistance (R) at maximal methacholine concentration (64 mg/ml) was increased in OVA challenged WT mice when compared to Sal treated WT mice (2.26 ± 0.21 vs. 1.69 ± 0.16, *p* = 0.03. Lung resistance was lower in both the saline and OVA-challenged β7-/- mice and there was no significant difference in response to OVA vs SAL in the knockout mice (β7-/- saline: 1.43 ± 0.17, β7-/- OVA 1.79 ± 0.07). Resistance was significantly reduced in β7-/- OVA as compared to WT-OVA mice (2.26.4 ± 0.21 vs 1.79 ± 0.07, *p* = 0.048). For total resistance measured by area under the curve (AUC) for all concentrations, AUC (mean ± SEM): WT-SAL 32.4 ± 6.1, β7-/- -SAL 25.11 ± 7.1, WT-OVA 44 ± 8.0, β7-/- -OVA 34.56 ± 5.2, *p* = 0.081 for WT-OVA vs β7-/- -OVA (Fig. 2B).

### Airway remodeling

We evaluated whether airway remodeling in response to acute and chronic OVA exposure is attenuated *in-vivo* in β7-/- mice. WT mice exposed to OVA showed an increase in goblet cell number, collagen content and airway smooth muscle as compared to SAL. OVA exposure in β7-/- mice was associated with reduced lung collagen deposition (ug/100mg lung tissue) compared to that in WT mice (WT acute 276.5 ± 53.3 vs β7-/- acute 196.1 ± 66.4, *p* = 0.021); WT chronic 262.1 ± 42 vs. β7-/- chronic 191.71 ± 32.6, *p* = 0.020) (Fig. 2C). There was increased airway (peribronchial) smooth muscle (ASM) in response to OVA in WT mice and this increase was attenuated in β 7-/- mice following both acute and chronic OVA exposure (WT-SAL 35.9 ± 3.02, β7-/- -SAL 39.4 ± 4.51; Acute: WT-OVA 60.6 ± 5.8 vs β7-/- OVA 31.6 ± 1, p = 0.03; Chronic: WT-OVA 101.7 ± 6.5 vs β7-/- OVA 48.5 ± 5.4, *p* = 0.03) (Fig. 2D). In OVA exposed β7-/- mice, no difference was observed in goblet cell number when compared to WT mice (Fig. 2E).

### TGF-β, IL-13

**Fig. 3:**
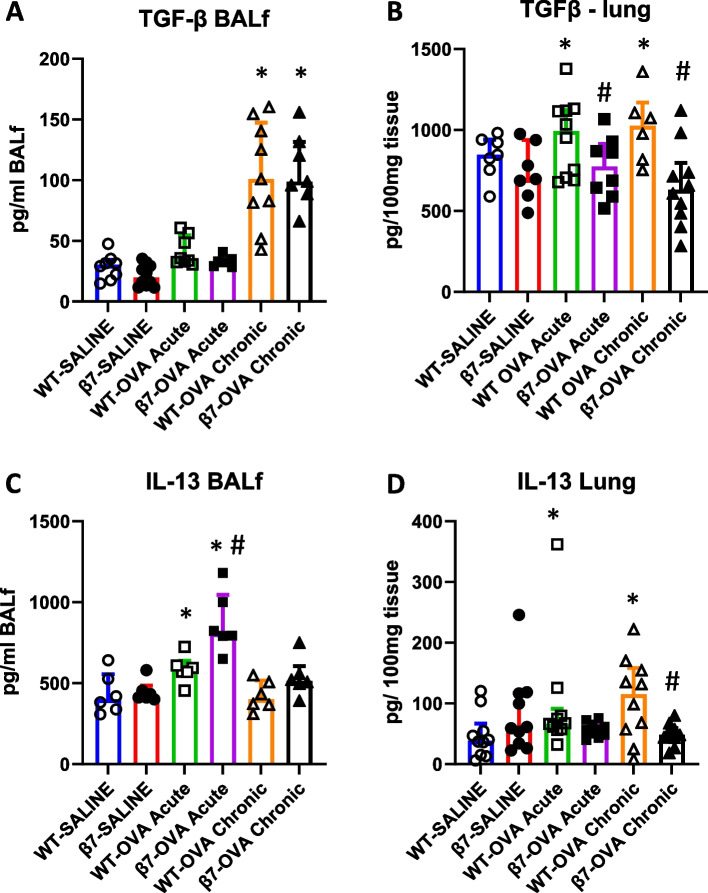
TGF β and Interleukin-13 expression in β 7-/- and WT mice treated with saline or OVA for one ("acute") or four weeks ("chronic"). **A** TGF β in BALf, **B** TGF β in lung tissue **C** IL-13 in BALf, **D** IL-13 in lung tissue. *p < 0.05 vs SAL, #*p* < 0.05 for β7 -/- OVA vs WT-OVA. *n* = 8–10 mice per group

Both TGF-β and IL-13 contribute to airway remodeling in asthma. TGF-β was increased in BAL fluid (BALf) in mice exposed to OVA for four weeks with no increase at one week. No difference was observed in BAL TGF-β expression between WT and β7**-/-** mice (Fig. 3A). In lung tissue, an increase in TGF-β was observed in both acute and chronic OVA exposure models with significant reduction in β7**-/-** mice as compared to wild type (WT-SAL769 ± 99 vs β7-/- SAL 737 ± 66 pg/100 mg tissue, acute: WT-OVA 1070 ± 72 vs β7-/- OVA 809 ± 71, *p* = 0.001; chronic: WT-OVA 1014 ± 89 vs β7-/- OVA 640 ± 63, *p* < 0.001) (Fig. 3B).

For IL-13, an increase was found in BAL fluid in mice exposed to OVA for one week, but not four weeks, with a greater increase in β7**-/-** mice than in WT (WT-SAL 436 ± 126 vs β7-/- SAL 449 ± 67 pg/ml BALf; acute: WT-OVA 587 ± 87, β7-/- OVA 873 ± 188, *p* = 0.04; chronic: WT-OVA 423 ± 91, β7-/- OVA 536 ± 118) (Fig. 3C). In lung tissue, an increase in IL-13 was observed in both acute and chronic OVA exposure models, with a significant reduction in β7**-/-** mice as compared to wild type in the chronic model only WT (WT-SAL 43 ± 3.4 vs β7-/- SAL 57 ± 3.8 pg/100 mg tissue; acute: WT-OVA 85 ± 12, β7-/- OVA 105 ± 18, NS; chronic: WT-OVA 74 ± 8, β7-/- OVA 52 ± 5, *p* < 0.05 (Fig. 3D).

### Anti α4β7 antibody

Based on the above findings in β7-/- mice, we wished to confirm the relevance of the α4β7 integrin receptor complex in OVA-induced AHR and remodeling in genetically "normal" (WT) mice. Wild type C57BL/6 mice were sensitized and then challenged with ovalbumin (OVA) or saline three times a week by inhalation for 4 weeks in the presence or absence of a neutralizing α4β7 antibody (DATK32, Southern Biotech, Alabama, USA) (100ug given intraperitoneally weekly from day 0, total of 6 doses) or intraperitoneal saline (Fig. 1).

**Fig. 4:**
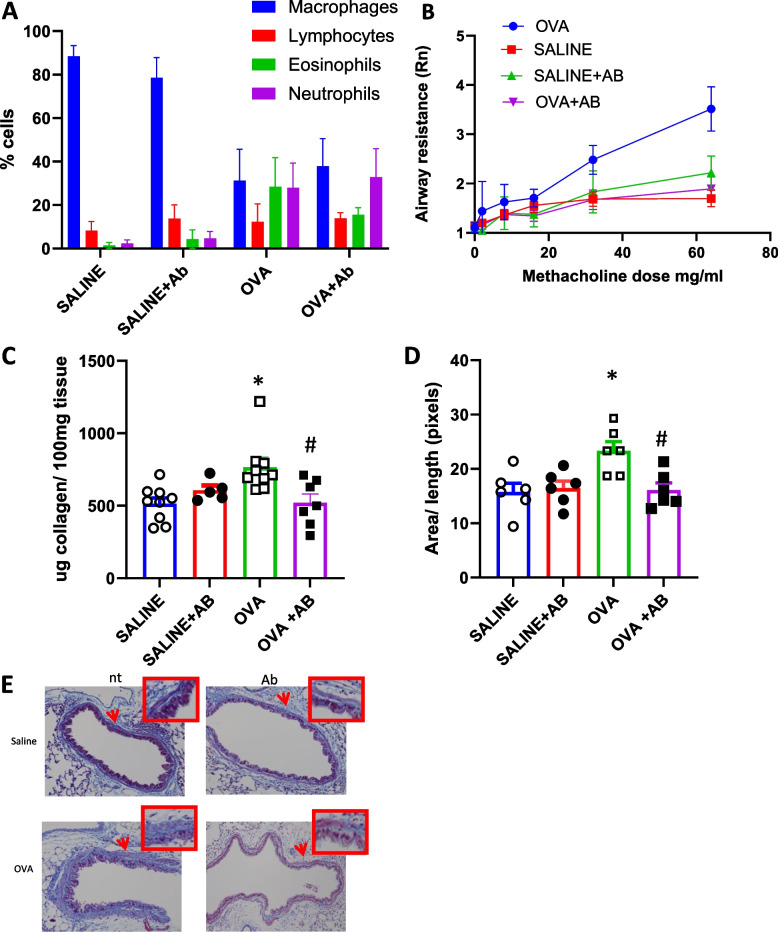
Bronchoalveloar cell counts, airway hyperresponsiveness, collagen and smooth muscle content in the presence or absence of neutralizing anti-α4β7 antibody. C57BL/6 mice were exposed to saline or OVA for 4 weeks in the presence or absence of neutralizing anti-α4β7 antibody (100ug in 100µl PBS given intraperitoneally weekly from day 0, total of 6 doses) or intraperitoneal saline. **A** Bronchoalveolar fluid cell counts (%). **B** Airway hyperresponsiveness (Airway / Newtonian resistance—Rn) in response to increasing doses of inhaled methacholine (MCH), *p* < 0.01 for area under the curve (AUC) and for resistance at the maximal MCH dose (64 mg/ml) for OVA vs OVA + antibody. **C** lung collagen content, *p < 0.01 for OVA vs SAL, # p = 0.014 for OVA vs OVA + antibody (**D**,** E**) Peribronchial smooth muscle in Masson Trichrome stained lung sections: (**D**) area expressed as pixel number using image J Pro Plus software. **E** representative histological images, nt: not treated with antibody. *p < 0.05 for OVA vs SAL, # *p* < 0.05 for OVA vs OVA + antibody. *n* = 5–8 for each group

The effect of α4β7 antibody on airway inflammation was similar to that observed in the β7-/- mice. There was an increase in total BAL cell counts in response to OVA exposure with an increase in eosinophils and neutrophils. The effect on inflammation in the presence of antibody suggests an increase in neutrophils relative to OVA, although this was not significant (Fig. 4A). Peribronchial tissue inflammation was also evaluated using semi-quantitative visual assessment in mice exposed to OVA or saline in the presence or absence of blocking α4β7 antibody. Inflammation is increased in response to OVA but was not diminished in the presence of antibody (data not shown).

Twenty-four hours after the last inhalation, AHR was measured using the flexivent ventilator system. Total lung resistance (R), airway resistance (Newtonian resistance, Rn) and tissue damping were all significantly increased in OVA challenged mice when compared to saline treated mice. There was complete abrogation of the airway hyper responsiveness in the OVA treated mice that received blocking α4β7 antibody and AHR was similar to that in the saline controls. For Rn, at maximal methacholine concentration (64 mg/ml), OVA 3.51 ± 0.36, OVA + antibody (Ab) 1.89 ± 0.05 (*p* < 0.01). Administration of antibody alone had no effect on AHR in the mice (SAL 1.69 ± 0.15, SAL + Ab 2.21 ± 0.24) (Fig. 4B). Area under the curve for the entire methacholine dose response showed increased AHR in OVA (90.54 ± 9.4) vs SAL treated mice (37.66 ± 4.02), which was inhibited in the presence of antibody (37.77 ± 5.40) (*p* < 0.01).

For parameters of remodeling, prolonged OVA exposure was associated with increased lung collagen deposition and this was reduced in mice treated with antibody (SAL: 514 ± 121ug collagen/100 mg lung tissue, OVA 765 ± 182, SAL + Ab 606 ± 73, OVA + Ab 520 ± 158; *p* < 0.01 for SAL vs OVA, *p* = 0.014 for OVA vs OVA + Ab) (Fig. 4C). There was an increase in airway (peribronchial) smooth muscle (ASM) in response to OVA and this increase was attenuated in mice treated with blocking α4β7 antibody (SAL 15.76 ± 1.3, OVA 23.3 ± 2.6, SAL + Ab 16.4 ± 2.7, OVA + Ab 16 ± 1.7, *p* < 0.05 for SAL vs OVA and for OVA vs OVA + Ab) (Fig. 4D,E). OVA exposed mice also have increased goblet cell number, which was only slightly and not significantly reduced in the mice that received antibody (data not shown).

### Fibroblast behavior in response to OVA in β7 -/- mice

**Fig. 5:**
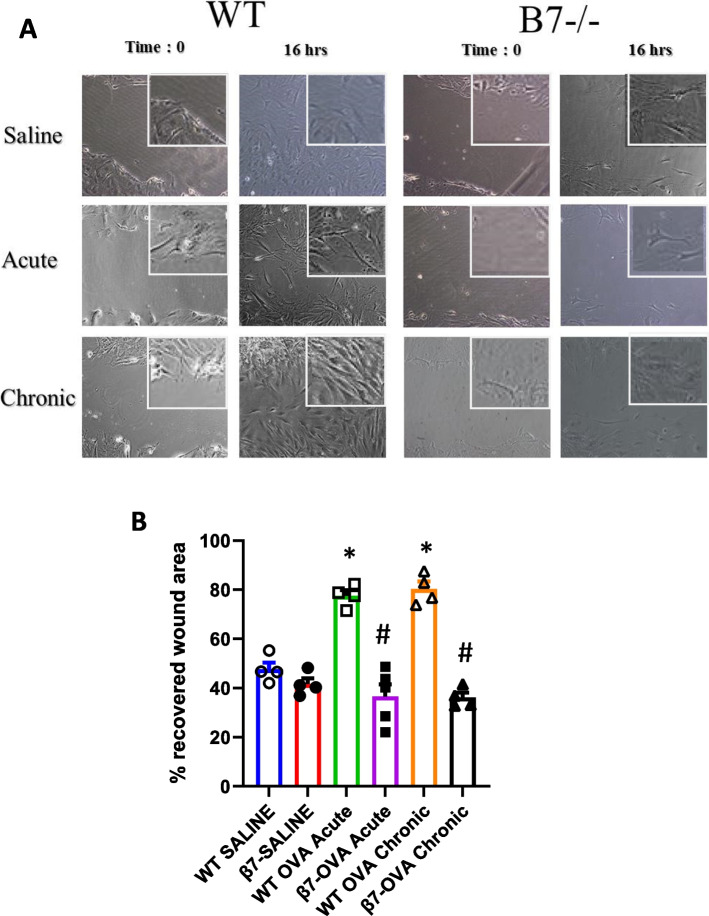
Lung fibroblast migration from WT and β7-/- mice. **A**, **B** Murine lung fibroblasts from WT and from β7-/- mice exposed to saline, OVA one week (acute) or OVA for 4 weeks (chronic exposure) were grown in 12-well culture plates until confluence. Following scratch wound, cell migration is expressed as percentage closure of wound at 16 h. **A** Representative images (20X and 50X (insert) (**B**) % recovered wound area. *p < 0.001 WT-OVA vs saline, *p < 0.001 β7-/- OVA vs WT-OVA, *n* = 4 per group

To determine whether fibroblast differentiation is altered in β7 -/- mice, lung fibroblasts from WT and β7 -/- mice exposed to saline or OVA for one (acute) or four weeks (chronic) were cultured ex-vivo. Using the wound scratch assay, fibroblast migration was greatly enhanced in response to OVA in WT fibroblasts but not in the β7 -/- fibroblasts at both the one week and four week time points WT (WT-SAL 47.6 ± 2.76 vs β7-/- SAL 41.6 ± 2.37% recovered wound area; Acute: WT-OVA 72.2 ± 5.60, β7-/- OVA 36.6 ± 4.92, *p* = 0.0014; Chronic: WT-OVA 80.3 ± 3.06, β7-/- OVA 36.2 ± 2.04, *p* < 0.001 (Fig. 5A,B). There was no difference in migration between WT and β7 -/- in the saline treated mice. β7 -/- fibroblasts had reduced proliferation in both saline and OVA treated mice as compared to WT mice (data not shown).

**Fig. 6:**
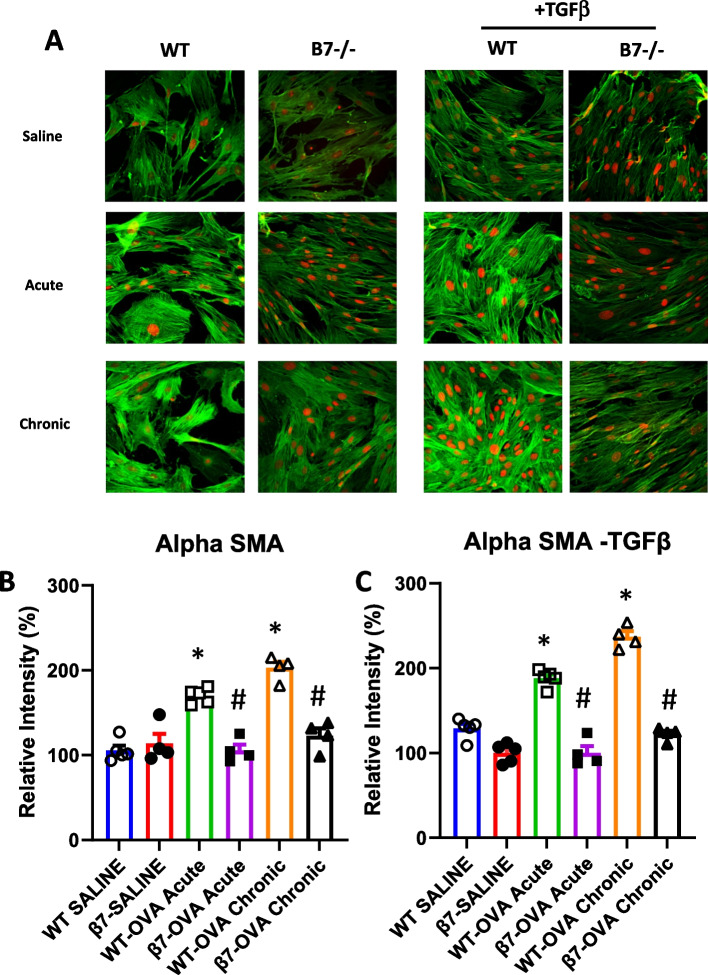
Expression of α-SMA in murine lung fibroblasts from WT and β7-/- mice. Fibroblasts (passage 2) from WT and from β7-/- mice exposed to saline, OVA one week (acute) or OVA for 4 weeks (chronic exposure) were grown in culture plates for 48 h in the presence or absence of TGF-β (10ng/ml). Cells were stained for α-smooth muscle actin (α-SMA) (Sigma Aldrich, USA) (FITC—green) and PI for nuclei (red) (**A**). Average intensity of fluorescence (expressed as mean % change density/ intensity relative to control mice (WT-Sal) is shown in **B** (without TGF-β) and** C** (with TGF-β). *N* = 4, (* *P* < 0.05 for WT SAL vs OVA, #*p* < 0.05 for. β7-/- OVA vs WT-OVA)

**Fig. 7:**
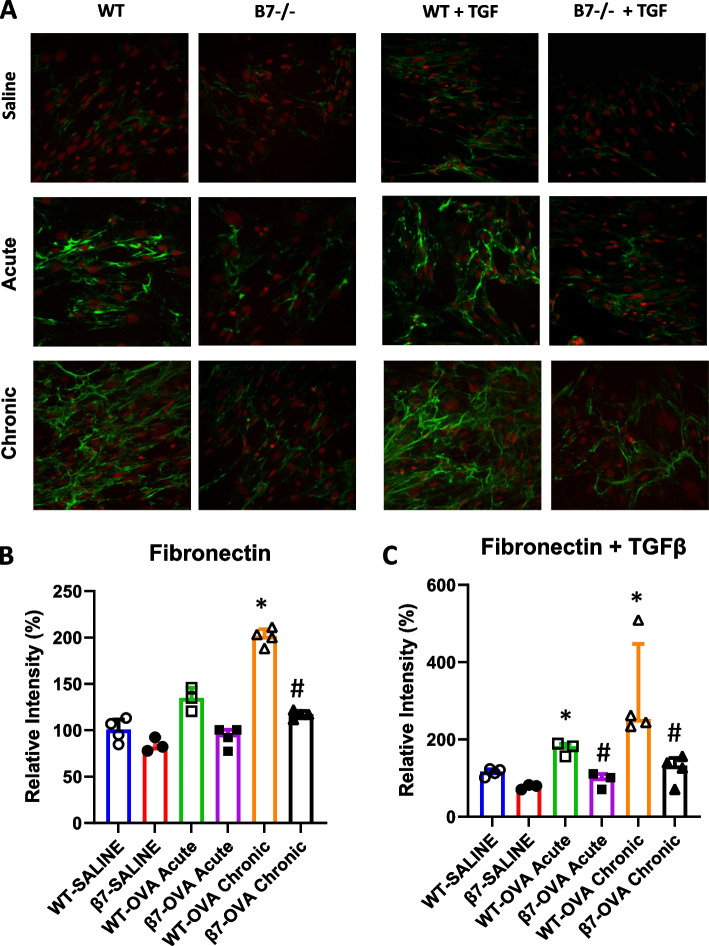
Expression of fibronectin in murine lung fibroblasts from WT and β7-/- mice. Murine lung fibroblasts (passage 2) from WT and from β7-/- mice exposed to saline, OVA one week (acute) or OVA for 4 weeks (chronic exposure) were grown in culture plates for 48h in the presence or absence of TGF-β (10ng/ml). Cells were stained for total fibronectin (3E2, Abcam, Cambridge, UK) (FITC—green) and PI for nuclei (red) (**A**). Average intensity of fluorescence (expressed as mean %Change Density/Intensity relative to control mice (WT-SAL) is shown in **B** (without TGF-β) and** C** (with TGF-β). *N* = 4, (* *P* < 0.05 for WT SAL vs OVA, #*p* < 0.05 for β7-/- OVA vs WT-OVA

To determine expression of fibroblast derived extracellular matrix proteins, lung fibroblasts were cultured to confluence in the presence or absence of TGFβ and cells were stained with immunoflourescent antibodies for α-smooth muscle actin (αSMA) (Fig. 6, Table 1) and for total fibronectin (Fig. 7, Table 1). TGFβ resulted in a modest increase in expression of αSMA and of total fibronectin relative to untreated fibroblasts. For both proteins, there was marked increase in expression in response to OVA at both one and four weeks and this was reduced in β7 -/- mice.

**Table 1:** Immunoflourescence staining of murine lung fibroblasts (relative to saline)

		No TGFβ		+ TGFβ	
		Wild type	β7 -/-	Wild type	β7 -/-
αSMA	Saline	100	102.2 ± 9.0	129.0 ± 15.9	98.3 ± 15.3
	OVA—acute	*170.0 ± 9.4	#105.5 ± 20.1	*188.1 ± 13.2	#92.3 ± 10.5
	OVA- chronic	*209.7 ± 15.8	#130.9 ± 13.3	*241.8 ± 19.8	#126.1 ± 11.8
Fibronectin	Saline	100	84.2 ± 13.4	114.7 ± 14.4	77.4 ± 6.3
	OVA—acute	131.2 ± 21.9	97.7 ± 8.0	*175.9 ± 19	#106.1 ± 14.1
	OVA-chronic	*200.7 ± 14.3	#114.3 ± 6.3	*239.3 ± 8.0	#140.8 ± 27.3

^*^p < 0.05 vs saline, #*p* < 0.05 vs wild-type. Mean ± SEM, *N* = 4

## Discussion

In this study, we demonstrate that the α4β7 integrin is necessary for the development of airway remodeling and airway hyperresponsivenss in a murine model of allergen-induced "asthma". Ovalbumin exposed integrin β7 receptor subunit null mice (β7-/-) have reduced airway hyperresponsiveness and reduced airway remodeling when compared to wild-type mice. We confirm these findings using a blocking α4β7 antibody in wild-type allergen-exposed mice. We also demonstrate that activation and differentiation of murine lung fibroblasts from β7 -/- mice in response to OVA is impaired.

The mechanism whereby targeting α4β7 or the β7 integrin receptor subunit reduced remodeling and airway hyperresponsiveness in our study is likely to be multifactorial. This effect may be via attenuation of inflammation, however, we found that airway and lung eosinophilia was not significantly altered in our mice. The α4β7 integrin receptor is present on a variety of inflammatory cells, particularly lymphocytes, eosinophils and mast cells [20, 21]. This integrin complex is expressed on murine eosinophils but, in contrast to gut eosinophils, is downregulated on lung and BAL eosinophils recruited in response to allergen exposure and there is no reduction in lung eosinophilia in allergen exposed β7 -/- mice [22]. Other studies have reported that the β7 integrin does mediate eosinophil recruitment to the lung in response to OVA or to parasite infection [27]. We observed an apparent increase neutrophil numbers in α4β7-antibody treated mice. The explanation and significance of this finding is unclear and needs to be explored in further studies. It is possible that blocking α4β7 on other inflammatory or immune cell types, such as specific lymphocyte subsets or mast cells may also contribute to impaired remodeling. α4β7 integrin expressed on gut lymphocytes contributes to the pathogenesis of inflammatory bowel disease via interaction with endothelial MAdCAM-1. Mast cell progenitor numbers are dramatically increased in the lungs of ovalbumin-sensitized mice and this recruitment is reduced in β7 deficient mice [28]. This effect was attributable to the α4β7 complex (in association with VCAM) as recruitment of mast cell progenitors was unaffected by αE blockade.

We found that targeting the α4β7 integrin complex and the β7 subunit impairs fibroblast activation in response to allergen exposure and believe this is likely to be an important factor in reducing remodeling. Increased numbers of fibroblasts / myofibroblasts have been reported in the airways of asthmatic subjects, as well as in mice, after chronic exposure to OVA [9, 10, 12, 29, 30] We have previously described that α4β7 binds fibronectin via the extra-domain-A segment (EDA-FN) and mediates EDA-FN induced fibroblast differentiation [11, 25]. Several other integrins, including α_5_β_1,_ α_4_β_1_ and α_9_β_1,_ are expressed on fibroblasts and contribute to lung fibrosis via interaction between fibroblasts and extra-cellular matrix [23, 24]. There may also be a role for the α4β7integrin on airway smooth muscle cells, although this remains speculative. Integrin mediated smooth muscle interaction has been investigated for other integrins and has been shown to mediate airway hyperresponsiveness and remodeling in a human asthma ASM cells and in a murine model of ovalbumin induced “asthma”. The proposed mechanisms are different for different integrins. Eosinophils bind to ASM via the RGD-binding integrins, α4β1 and αMβ2, and increase ASM cell proliferation and TGFβ1, collagen and fibronectin expression [31]. αvβ6 blockade alters expression of murine chymase, α5β1 blockade interferes with fibronectin-ASM interaction and inhibition of α2β1 impairs collagen and laminin binding with ASM [32, 33]. These studies emphasize the importance of extra-cellular matrix -integrin- airway smooth muscle interaction in the development of AHR. Silencing or blocking of the β1 integrin using a shRNA or a α5β1/ αvβ1 dual antagonist in mice has been shown to reduce airway remodeling / hyperresponsiveness in response to OVA [34, 35].

In our study, airway hyperresponsiveness "tracked" with remodeling rather than with inflammation. The major factors contributing to AHR in asthma are still open to debate, Asthmatic patients receiving optimal anti-inflammatory treatment show little or no change in AHR [36]. Several studies have documented a correlation between AHR in asthmatic patients and the presence of subepithelial fibrosis [37, 38] and ECM protein deposition by activated bronchial fibroblasts [39]. Asthma severity and reduced lung function were found to correlate most closely with airway fibroblast accumulation and ASM hypertrophy [30]. In murine models, some studies suggest that airway fibrosis per se might be an important factor contributing to AHR, whereas others found no such relationship [39, 40]. As mentioned above, airway hyperresponsiveness is attenuated in murine models of asthma (and in human tracheal rings) without altering airway inflammation when integrin mediated tethering of ECM proteins to airway smooth muscle cells is blocked.

We used an OVA model of allergen-induced murine “asthma”. Using a house-dust mite model, which may more closely approximate human asthma, could have given different results, however, we think this is unlikely.

Airway remodeling was long considered a consequence of inflammation but it has become apparent that it may occur as a primary event independent of inflammation [41–44]. AR is observed in children with asthma [45] and may be related to lung development, and epigenetic factors [46–51]. There are few studies demonstrating that remodeling can be modified in asthmatic patients and there are no approved medications for this indication. It is also not known whether treating or attenuating remodeling will reduce AHR in asthmatic patients. Corticosteroids, the cornerstone of asthma therapy, have pleiotropic anti-inflammatory effects, but their efficacy in attenuating remodeling is poorly established [52]. There is some evidence that biologicals used in severe asthma may reduce remodeling, with most data available for omalizumab [53]. Bronchial thermoplasty reduces frequency and severity of asthma exacerbations but its effect of AHR is not clear. Although this technique is thought to target submucosal ASM, it may also act on nerve endings in the airways.

There are several drugs available that inhibit integrins and may reduce variable features of asthma. An oral α4β1/α4β7 dual antagonist reduced inflammation and AHR in rats [54]. The non-selective a4 neutralizing antibody natalizumab and the selective α4β7 antibodies vedolizumab and abrilumab as well as the β7 specific antibody, etrolizumab have shown efficacy in the treatment of inflammatory bowel disease. Oral small molecule inhibitors of α4β7 are also being evaluated for IBD [55, 56].

In conclusion, we demonstrate that the β7 integrin subunit and the α4β7 integrin complex modulate airway hyperresponsiveness and airway remodeling. This effect is, at least in part, explained by inhibition of fibroblast activation / differentiation and is not clearly related to eosinophilic inflammation. Our study suggests that selective inhibition of this integrin may serve as a novel therapeutic option to attenuate the development of airway fibrosis and AHR in patients with asthma.

### Supplementary Information

Supplementary Material 1.

## Data Availability

No datasets were generated or analysed during the current study.

## Abbreviations

AHR: Airway hyperresponsiveness
AWR: Airway remodeling
αSMA: Alpha-smooth muscle actin
ASM: Airway smooth muscle
BAL: Bronchoalveolar lavage
BALf: Bronchoalveolar lavage fluid
IL-13: Interleukin-13
ECM: Extra-cellular matrix
EDA: Extra-domain A
OVA: Ovalbumin
SAL: Saline
TGF β: Transforming growth factor beta
WT: Wild-type
