# Supplementary material for: The integrin receptor beta7 subunit mediates airway remodeling and hyperresponsiveness in allergen exposed mice
Source: Respir Res. 2024 Jul 12;25:273. doi: 10.1186/s12931-024-02899-8 (PMC11241790; doi:10.1186/s12931-024-02899-8)
Supplement: Supplementary file 1 — Supplementary Material 1. [file 12931_2024_2899_MOESM1_ESM.zip › Legend Supplementary Figure 1.docx]

Legend Supplementary Figure 1

PCR was performed on DNA obtained from the tail of β7-/- mice using primers purchased from Jackson Laboratory ( and according to the “Touchdown cycling protocol” as recommended by Jax Laboratory) . (Bar Harbour, NE, USA)

<https://www.jax.org/Protocol?stockNumber=002965&protocol>, ID=25367

B7 primers: Beta7-F: GGAGGCATACTGTGCCAACT, Beta7-R: CCCTCCTTCAGGACTGACAC

Product size: 170 bp

Lane 1 size marker, Lane 2,3 WT mice, lanes 4,5 β7-/-mice, lane 6: no template control (NTC).
