# Supplementary figures and images for: The integrin receptor beta7 subunit mediates airway remodeling and hyperresponsiveness in allergen exposed mice
Source: Respir Res. 2024 Jul 12;25:273. doi: 10.1186/s12931-024-02899-8 (PMC11241790; doi:10.1186/s12931-024-02899-8)

## Slide 1
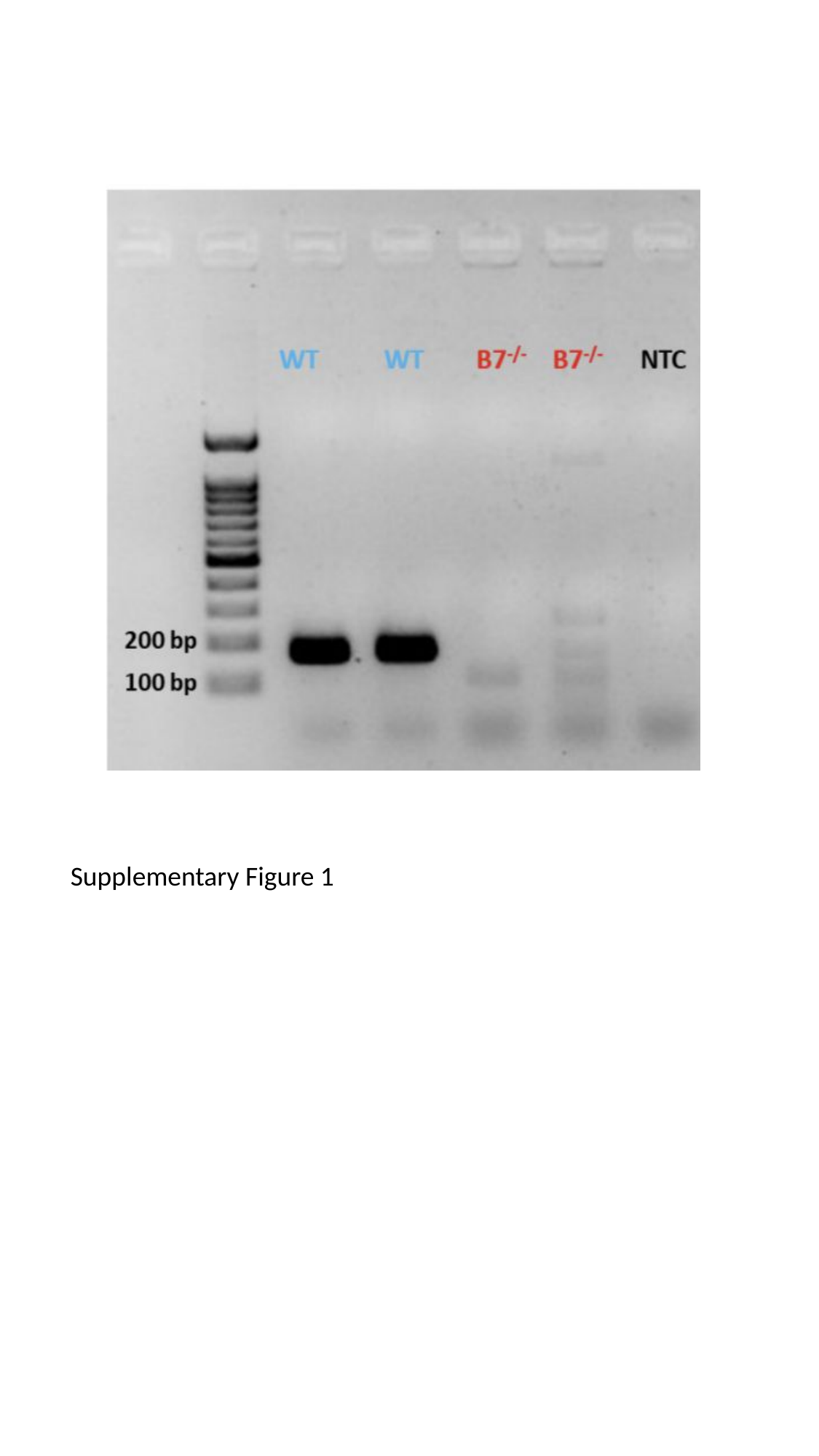

Supplementary Figure 1

Supplement: Supplementary file 1 — Supplementary Material 1. [file 12931_2024_2899_MOESM1_ESM.zip › RESP RES Supplementary Figure 1.pptx]
